# Brain signal complexity in adults with Down syndrome: Potential application in the detection of mild cognitive impairment

**DOI:** 10.3389/fnagi.2022.988540

**Published:** 2022-10-20

**Authors:** Alberto Fernández, Federico Ramírez-Toraño, Ricardo Bruña, Pilar Zuluaga, Susanna Esteba-Castillo, Daniel Abásolo, Fernando Moldenhauer, Elizabeth Shumbayawonda, Fernando Maestú, Javier García-Alba

**Affiliations:** ^1^Department of Legal Medicine, Psychiatry and Pathology, Universidad Complutense de Madrid, Madrid, Spain; ^2^Institute of Sanitary Investigation (IdISSC), Hospital Universitario San Carlos, Madrid, Spain; ^3^Center for Cognitive and Computational Neuroscience, Universidad Complutense de Madrid, Madrid, Spain; ^4^Department of Experimental Psychology, Cognitive Processes and Speech Therapy, Universidad Complutense de Madrid, Madrid, Spain; ^5^Department of Radiology, Universidad Complutense de Madrid, Madrid, Spain; ^6^Department of Industrial Engineering & IUNE & ITB, Universidad de La Laguna, San Cristóbal de La Laguna, Spain; ^7^Statistics & Operations Research Department, Faculty of Medicine, Universidad Complutense de Madrid, Madrid, Spain; ^8^Neurodevelopmental Group, Girona Biomedical Research Institute-IDIBGI, Institute of Health Assistance (IAS), Parc Hospitalari Martí i Julià, Girona, Spain; ^9^Centre for Biomedical Engineering, School of Mechanical Engineering Sciences, University of Surrey, Guildford, United Kingdom; ^10^Adult Down Syndrome Unit, Internal Medicine Department, Health Research Institute, Hospital Universitario de La Princesa, Madrid, Spain; ^11^Department of Research and Psychology in Education, Universidad Complutense de Madrid, Madrid, Spain

**Keywords:** Down syndrome, magnetoencephalography, brain signal complexity, mild cognitive impairment, neuropsychological performance, adult

## Abstract

**Background:**

Down syndrome (DS) is considered the most frequent cause of early-onset Alzheimer’s disease (AD), and the typical pathophysiological signs are present in almost all individuals with DS by the age of 40. Despite of this evidence, the investigation on the pre-dementia stages in DS is scarce. In the present study we analyzed the complexity of brain oscillatory patterns and neuropsychological performance for the characterization of mild cognitive impairment (MCI) in DS.

**Materials and methods:**

Lempel-Ziv complexity (LZC) values from resting-state magnetoencephalography recordings and the neuropsychological performance in 28 patients with DS [control DS group (CN-DS) (*n* = 14), MCI group (MCI-DS) (*n* = 14)] and 14 individuals with typical neurodevelopment (CN-no-DS) were analyzed.

**Results:**

Lempel-Ziv complexity was lowest in the frontal region within the MCI-DS group, while the CN-DS group showed reduced values in parietal areas when compared with the CN-no-DS group. Also, the CN-no-DS group exhibited the expected pattern of significant increase of LZC as a function of age, while MCI-DS cases showed a decrease. The combination of reduced LZC values and a divergent trajectory of complexity evolution with age, allowed the discrimination of CN-DS vs. MCI-DS patients with a 92.9% of sensitivity and 85.7% of specificity. Finally, a pattern of mnestic and praxic impairment was significantly associated in MCI-DS cases with the significant reduction of LZC values in frontal and parietal regions (*p* = 0.01).

**Conclusion:**

Brain signal complexity measured with LZC is reduced in DS and its development with age is also disrupted. The combination of both features might assist in the detection of MCI within this population.

## Introduction

Down syndrome (DS) is a genetic condition linked to the overexpression of chromosome 21, and has been considered the most frequent cause of early onset Alzheimer’s disease (AD) associated with a specific genotype (McCarron et al., 2017; Chen et al., 2018). In spite of this, the number of studies on the characterization of predementia stages, such as mild cognitive impairment (MCI), is scarce, probably due to the inherent difficulties for the diagnosis of this condition (Mak et al., 2017; Esteba-Castillo et al., 2022). Currently, there is no official international consensus that delimits the characterization of MCI in DS. Esteba-Castillo et al. (2022) proposed diagnostic criteria for MCI in DS. Although there are publications trying to characterize MCI in DS, this aspect is complicated given the enormous cognitive variability and the intellectual disability typical in people with DS.

Considering these difficulties, some noninvasive evaluation techniques such as the electroencephalography (EEG) or magnetoencephalography (MEG) have been used. DS and AD share some basic neurophysiological characteristics, such as the general slowing of EEG or MEG oscillations including a reduction of alpha power and peak frequency (Katada et al., 2000; Menéndez, 2005; Hamburg et al., 2019). However, the lack of neurophysiological investigations focused on MCI in DS is also notable, as most published studies compared groups of young or middle-aged DS patients with age-matched controls.

In the last few decades, novel techniques evaluating the complexity of brain signals have been added to the conventional power spectrum analysis. Complexity estimators are suitable methods to quantify the linear or nonlinear modifications of brain activity over time (for a review, see Stam, 2005). Different complexity estimators have been utilized to investigate and assist in the diagnosis of medical and neuropsychiatric disorders, and particularly to assess the brain’s oscillatory changes appearing in the AD-spectrum (for reviews see Fernández et al., 2013b; Sun et al., 2020). These studies reported a profile of reduced complexity in AD and MCI patients. Lempel–Ziv complexity (LZC) is an easy to compute algorithm evaluating the complexity in a time series by identifying the number of distinct substrings and the rate of their occurrence along a temporal sequence (Lempel and Ziv, 1976). LZC is dependent on the bandwidth of the signal spectrum, and therefore brain signals displaying a broader spectrum would yield higher values of complexity (Aboy et al., 2006).

Based on the link between AD and DS, it might be expected that signals recording brain activity in DS would be less complex than those from healthy controls, but current evidence seem to contradict such assumption, perhaps due to the age-interval of the samples. For instance, Hemmati et al. (2013) studied EEG signals by means of fractal dimension in a sample of children with DS, and results indicated that patients with DS showed higher complexity values than controls. Such tendency was confirmed in fMRI studies focused on the estimation of complexity values in young adults (Carbó-Carreté et al., 2020; Figueroa-Jimenez et al., 2021).

In this study we hypothesize that: (1) given the pathophysiological similarities between AD and DS, brain signals from adults with DS will show reduced signal complexity, with an additional reduction in the MCI group that might assist in the characterization of this stage; and (2) patients with DS, particularly those presenting MCI, would represent another example of abnormal changes of complexity with aging [i.e., they would not follow the clear pattern of complexity increase as a function of age within the healthy population previously found with EEG and MEG, as it was systematically “broken” in different pathologies (Anokhin et al., 2000; Fernández et al., 2012)]. In the present study, LZC was estimated from MEG signals, but taking advantage of MEG’s technical particularities, LZC values were calculated employing a source-based methodology (Shumbayawonda et al., 2019). Finally, it is important to emphasize that this piece of work is an extension of previous studies (García-Alba et al., 2019; Ramírez-Toraño et al., 2021) where some other markers were utilized to investigate the particular characteristics of MCI in DS.

## Materials and methods

### Participants

We recruited 28 DS participants (12 males and 16 females, mean age 47.21 ± 4.10 years) with genetically confirmed karyotype, excluding cases of mosaicisms and translocations, from the Unidad de Adultos con Síndrome de Down (Adult Down Syndrome Unit-La Princesa University Hospital, Madrid, Spain). This sample was divided into two groups: (1) patients with DS who did not match the criteria for MCI or AD diagnosis (CN-DS group) (4 males and 10 females, age 44.64 ± 3.29; min = 40, max = 52), and (2) patients with DS who matched the criteria for MCI (MCI-DS group) (8 males and 6 females, age 51.64 ± 3.96; min = 47, max = 61). In addition, we recruited a group of healthy control individuals (CN-no-DS) (4 males and 10 females, mean age 45.21 ± 4.38; min = 40, max = 54) free of any significant medical, neurologic, and/or psychiatric disease, and age and sex matched with the CN-DS group. The demographic characteristics of the sample are presented in Table 1.

**TABLE 1:** Demographic characteristics.

	CN-no-DS	CN-DS	MCI-DS	
	Mean (SD)	Mean (SD)	Mean (SD)	*P*-value (statistic)
n	14	14	14	
Age	45.214 (4.388)	44.643 (3.296)	51.643 (3.954)	<0.001 (13.870)
Sex	10 F / 4 M	10 F / 4 M	6 F / 8 M	0.199 (3.231)

The comparison of age was addressed by means of ANOVA. The comparison of sex was addressed by means of chi-square test.

Inclusion and exclusion criteria of the sample were exhaustively described in García-Alba et al. (2019). The study was conducted in accordance with The Code of Ethics of the World Medical Association (Declaration of Helsinki), and the protocol was approved by the Clinical Research Ethical Committee of La Princesa University Hospital. Written informed consent was obtained from parents or legal guardians, and verbal or written assent was additionally obtained from DS patients.

### Clinical assessment

An exhaustive clinical and neuropsychological assessment protocol was applied to all patients with DS. This protocol was not applied to CN-no-DS individuals.

Protocol:

- Cambridge Cognitive Examination for older adults with Down’s Syndrome (CAMCOG-DS) Spanish version (Esteba-Castillo et al., 2013). The CAMCOG-DS is a section of the CAMDEX-DS, dedicated to the cognitive assessment of the patient, which evaluates the main cognitive domains (orientation, language, memory, praxis, abstract thinking, attention, perception). The cognitive profile obtained by the CAMCOG-DS complements the diagnosis obtained by the CAMDEX-DS. The assessment consists of three parts: clinical interview, neuropsychological assessment and interviewer observations.
- Cambridge Examination for Mental Disorders of Older People with Down’s Syndrome and Others with Intellectual Disabilities (CAMDEX-DS) (Ball et al., 2006), Spanish version (Esteba-Castillo et al., 2013). Test adapted and validated for people with DS and other intellectual disabilities. The CAMDEX-DS assesses and diagnoses mental disorders and the impairment caused by AD disease, in our case we used the version adapted to Spanish. It consists of a structured interview administered to the informant/family member (CAMDEX-DS interview) and carried out in the absence of the patient. Its aim is to facilitate the systematic collection of the relevant symptomatology. It consists of four parts: best level of functioning of the patient/participant, cognitive and functional impairment, mental health and physical health.
- Barcelona Test-Intellectual Disability (BT-ID) (Esteba-Castillo et al., 2017). Neuropsychological test designed and validated in the Spanish population for adults with intellectual disabilities. This test is not diagnostic, it provides the complete cognitive profile of the person, and like the CAMCOG-DS, it is an excellent complement to the diagnosis provided by the CAMDEX-DS. The BT-ID is composed of 67 subtests related to eight cognitive domains: orientation, attention, working memory, language, praxis, memory, executive functions and visuoconstruction.
- Behavior Rating Inventory of Executive Function-Parents (BRIEF-P) (Gioia et al., 2010). We also included informant’s reports from the BRIEF-Informant’s Report. The BRIEF-P measures seven different aspects of executive function grouped into two indexes: The Behavioral Regulation Index is composed of inhibit, shift and emotional control; the Metacognitive Index is composed of initiate, working memory, planification, organization and monitor. We use this test as an extension of the BT-ID for the behavioral assessment of executive functions.

All the tests present a quantitative assessment method, however, and especially in the CAMDEX-DS, CAMCOG-DS, and BT-ID tests, it is very important to observe how the patient responds to each of the subtests (response times, perception of errors, etc.). From the tests aforementioned, only a few variables were selected for the statistical analyses: *BRIEF-GEC* (*global executive composite index* of the BRIEF); *working memory, verbal short-term memory-learning, verbal short-term memory-learning_delayed* and *constructive praxis* (BT-ID); *praxis total* (CAMCOG-DS). The CAMDEX-DS was only used as a diagnostic tool.

The diagnosis of MCI was based on expert clinical judgment, as it is recommended in the standard practice for DS (Sheehan et al., 2015; Fenoll et al., 2017; Pujol et al., 2018; Esteba-Castillo et al., 2022). For an MCI diagnosis, patients should present: (1) a report of cognitive impairment by the patient (confirmed by a reliable informant), or a report of cognitive impairment by a reliable informant implying a change from previous capacities; paired with (2) no clinically relevant deterioration in adaptive skills according to CAMDEX-DS informant section, were required (García-Alba et al., 2019; Ramírez-Toraño et al., 2021).

All participants were clinically assessed three times over a 3-year follow-up period. Initiation of the study (L_0_), 1 year after the start of the study (L_1_), and 2 years after the start of the study (L_2_). On each assessment, CAMDEX-DS was applied for follow-up diagnosis purposes. In L_0_ a clinical/neuropsychological study and MEG were performed, in L_1_ a clinical/neuropsychological study was conducted and in L_2_ the assessment included a clinical/neuropsychological study and MEG recording. Only results from L_0_ are presented in this article. The longitudinal nature of the study is mentioned, as in L_0_ the baseline condition of the patient was established and the follow-up served to confirm the diagnosis over time. Establishing the patient’s baseline status in patients with DS is fundamental given the cognitive deficit inherent to this syndrome; this allows us to differentiate between these cognitive deficits and those due to MCI impairment.

### Magnetoencephalography recordings and preprocessing

We acquired 4 min of eyes-closed resting-state activity at the Center for Biomedical Technology (Madrid, Spain) using an Elekta Vectorview system (102 magnetometers and 204 planar gradiometers). We used four head positioning coils to continuously determine the head position, and two electrodes to record the ocular activity. The coil position and the participant’s head shape were digitalized using a Fastrack Polhemus system. During the recordings, participants sat inside a shielded room and were instructed to keep still and relax. DS patients’ recordings were performed by an expert on intellectual disabilities (JGA) and were well tolerated by all patients.

Data were anti-alias filtered (0.1–330 Hz) and digitized with a sampling frequency of 1,000 Hz. The spatio-temporal signal space separation (tSSS) method (Taulu and Simola, 2006), as implemented by MaxFilter (version 2.2, correlation 0.90, time window 10s), was used to remove external noise and compensate for head movements inside the MEG scanner. The recordings were inspected automatically for artifacts using the FieldTrip (Oostenveld et al., 2011), and the result was confirmed by an MEG expert (FRT). Data were segmented in non-overlapping 4-s epochs of continuous artifact-free data and at least 20 epochs were obtained from each recording. Finally, as the information contained in the MEG data is highly redundant after tSSS (Garcés et al., 2017), only magnetometers’ information was selected for analysis.

### Source reconstruction

We defined the source model based on a 1 cm homogeneous grid in MNI space and labeled each source according to the Harvard-Oxford atlas. The source model was linearly transformed into individual space using the individual head shape, and a lead field was estimated using a realistic single shell model based on the transformed MNI template (Nolte, 2003). We reconstructed the source-space time series using a Linearly Constrained Minimum Variance (LCMV) beamformer using the epoch-averaged covariance matrix.

### Lempel-Ziv complexity

LZC calculates the complexity of a signal in Kolmogorov’s sense after it has been coarse-grained into a finite symbol sequence. To this end, first we concatenated all the 4-s epochs from each source position *i*, resulting in a time series *x*_*i*_(*n*). Then, *x*_*i*_(*n*) was converted into a binary sequence [*s*_*i*_(*n*)] using the median as a threshold _(T_d_)_ (Fernández et al., 2012; Abásolo et al., 2015). This coarse-graining process improves the robustness to arbitrary outliers and ensures an equal number of zeros and ones, thus reducing the possible bias in LZC (Aboy et al., 2006). This binary string was then scanned from left to right, and a complexity counter *c*_*i*_ was increased by one unit every time a new subsequence of consecutive characters was encountered. Last, we normalized *c*_*i*_ using its upper bound *b*_*i*_ (Aboy et al., 2006; Fernández et al., 2012), to obtain the LZC *C*_*i*_:


(1) $$C_{i}=L\,Z\,C=\frac{c_{i}}{b_{i}}$$

Normalized LZC values are in the range 0≤*LZC*≤1, where a value of 0 shows a stationary signal with no varying dynamics, and 1 shows a very complex signal with multiple complex dynamics (Aboy et al., 2006). LZC values were obtained for each source position *x*_*i*_(*n*). Since this investigation followed a source-based approach, the LZC values of the corresponding time series were averaged in anatomical areas of interest according to the Harvard-Oxford atlas, namely: left and right frontal lobe (LF_LZC and RF_LZC), left and right parietal lobes (LP_LZC and RP_LZC), left and right temporal lobes (LT_LZC and RT_LZC), and left and right occipital lobe (LO_LZC and RO_LZC).

### Statistical analyses

The demographic characteristics of the sample were compared by means of ANOVA for age and chi-square for sex. The results of each test are presented in Table 1.

Regarding LZC, first, we examined the difference in LZC scores across groups by means of ANOVA test and the *post-hoc* comparisons were addressed using the Dunnett’s *post-hoc* test. Variables showing significant differences among groups were submitted as “candidate variables” to logistic regression analyses. In addition, we examined if demographic variables such as age and sex modulate the LZC values within the groups. Sex was addressed by two-sample *t*-test and the Levene’s test to assess the homogeneity of variance. The possible age effect on LZC scores was addressed by estimating the Pearson correlation coefficient of these two variables for each group to avoid false correlations led by inter-groups differences (Makin and de Xivry, 2019). Furthermore, a linear regression analysis between age and LZC scores was performed and the equality of regression slopes between groups was tested by means of F-tests. The purpose of the analyses regarding the “ag was: (1) to assess the distinctive developmental patterns of the LZCs with age between groups, and (2) to confirm the need to include age in the logistic regression analyses.

Secondly, we evaluated the potential capability of LZC to assist in the discrimination of MCI cases in DS. For this purpose, multivariate logistic regression analyses were applied to discover which candidate variables (see above) may discriminate between groups (CN-DS vs. CN-no-DS and CN-DS vs. MCI-DS). The model’s performance was assessed by means of classification tables, along with Hosmer and-Lemeshow goodness of fit tests and Nagelkerke R^2^ coefficient. Hosmer-Lemeshow’s model-building procedure (Hosmer et al., 2013) was followed to obtain the final multivariate models. In this procedure, the selection process should begin with a careful univariate analysis of each variable. Upon completion of univariate analyses, variables for multivariate analysis are selected. Any variable whose univariate test is significant should be considered as a candidate for the multivariate model along with all variables of known biological importance. All candidate variables are submitted to a Forward Likelihood ratio method to select variables that should be included in the final model. Following the fit of the multivariate model, the interaction terms are tested. In addition, Receiver Operating Characteristic (ROC) curves and the goodness-of-fit tests are used to evaluate the precision of the final models.

Finally, the relationship between the LZC values and neuropsychological performance was assessed using the Spearman’s correlation coefficient due to the characteristics of neuropsychological scores. SPSS Statistics version 27.0 and Statgraphics version 19 were used for the statistical analyses, and a significance level of p < 0.05 was adopted for all comparisons. All *p*-values were two-tailed.

## Results

### Lempel-Ziv complexity differences between groups

**FIGURE 1:**
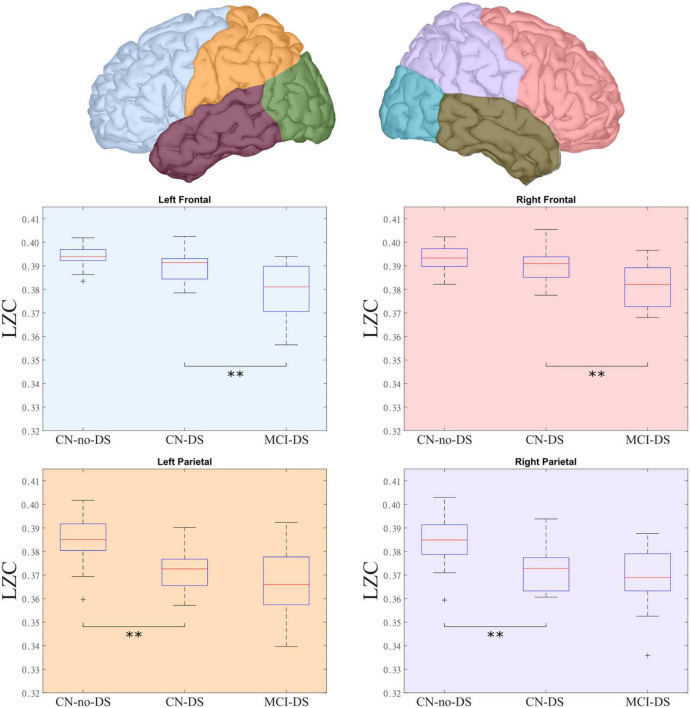
LZC distribution across groups and lobes. The top panel presents the lobes that presented significant differences in complexity among the groups. The bottom panel details these differences, showing the groups with significant differences in each lobe. The MCI-DS group showed the lowest complexity values, while the CN-DS group exhibited intermediate scores when compared with the CN-no-DS group. CN, control; DS, Down syndrome; LZC, Lempel-Ziv complexity; MCI, mild cognitive impairment. **Statistically significant difference between two groups.

The analysis showed significant differences between CN-no-DS and CN-DS groups in both LP_LZC (*p* = 0.037) and RP_LZC (*p* = 0.024), with smaller values in the CN-DS group. Moreover, a significant difference was found between CN-DS and MCI-DS groups in LF_LZC (*p* = 0.009) and RF_LZC (*p* = 0.014), with smaller values in the MCI-DS group. Results are presented in Table 2 and in Figure 1. Based on these results, LP_LZC, RP_LZC, LF_LZC, and RF_LZC scores were selected as candidate variables for the logistic regression models (see below). Additionally, regardless of the diagnostic group, frontal regions displayed the highest mean values as compared with the other anatomical regions.

**TABLE 2:** LZC values across groups and areas.

	CN-no-DS	CN-DS	MCI-DS	ANOVA	CN-DS vs. CN-no-DS	CN-DS vs. MCI-DS
	Mean (SD)	Mean (SD)	Mean (SD)	*P*-value	*P*-value	*P*-value
LF_LZC	0.394 (0.005)	0.387 (0.007)	0.380 (0.012)	<0.001	0.314	0.009
RF_LZC	0.393 (0.006)	0.390 (0.007)	0.382 (0.010)	0.001	0.380	0.014
LP_LZC	0.383 (0.011)	0.373 (0.009)	0.366 (0.014)	0.002	0.037	0.270
RP_LZC	0.384 (0.012)	0.372 (0.010)	0.368 (0.011)	0.003	0.024	0.541
LT_LZC	0.382 (0.011)	0.376 (0.008)	0.368 (0.017)	0.027	0.436	0.176
RT_LZC	0.383 (0.011)	0.376 (0.010)	0.369 (0.009)	0.009	0.243	0.147
LO_LZC	0.369 (0.015)	0.363 (0.014)	0.362 (0.016)	0.423	0.486	0.971
RO_LZC	0.368 (0.016)	0.363 (0.015)	0.360 (0.016)	0.420	0.595	0.869

*P*-value for ANOVA and *post-hoc* (Dunnett’s test) resulting from the comparison between groups. CN, control; DS, Down syndrome; LF, left frontal; LO, left occipital; LP, left parietal; LT, left temporal; LZC, Lempel-Ziv complexity; MCI, mild cognitive impairment; RF, right frontal; RO, right occipital; RP, right parietal; RT, right temporal; SD, standard deviation.

### Demographic variables that modulate Lempel-Ziv complexity values

**FIGURE 2:**
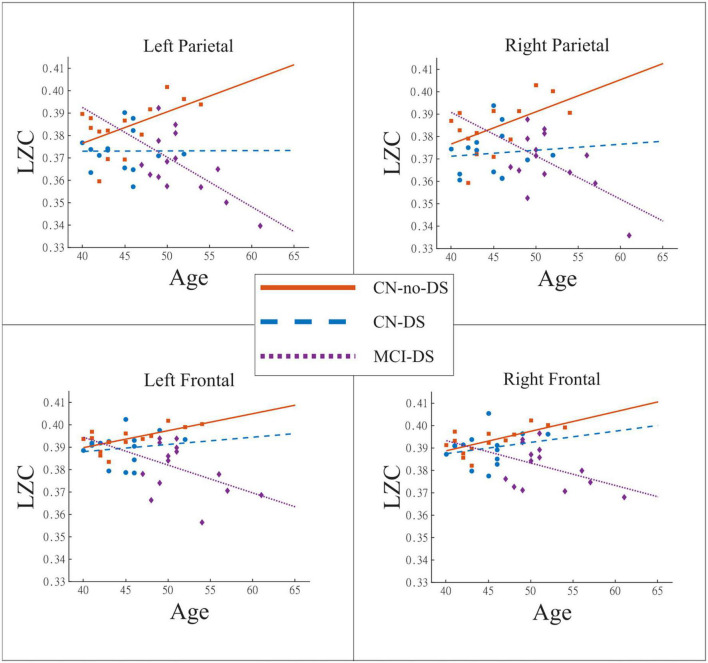
Relationship between Age and LZC. These plots use regression lines to show the evolution of the brain signals complexity with the age of the participant. The CN-no-DS group exhibited the expected pattern of significant increase of LZC as a function of age, while that pattern was “broken” in CN-DS and especially in MCI-DS. CN, control; DS, Down syndrome; LZC, Lempel-Ziv complexity; MCI, mild cognitive impairment.

Overall, the analyses demonstrated a pattern of positive correlations between age and LZC values in CN-no-DS and CN-DS groups, and negative correlations in the MCI-DS group. Such pattern was significant in the CN-no-DS group for LF_LZC (*p* = 0.014), RF_LZC (*p* = 0.009), LP_LZC (*p* = 0.048), and RP_LZC (*p* = 0.049) regions. Significant negative correlations were found in the MCI-DS group for LP_LZC (*p* = 0.017) and RP_LZC (*p* = 0.035). No significant correlations emerged in the CN-DS group. Moreover, significant differences were found when the slopes of the regression lines between age and LZC values were compared (*p* < 0.05 for all regions except for LT_LZC, see Table 3 and Figure 2). These results indicate that at least the slope of one group is significantly different from the rest. This tendency is reinforced by the previously described negative correlation between age and LZC in the MCI-DS group, and the significantly higher mean of age in the MCI-DS group (*p* < 0.01). Consequently, age will be included in all the classificatory logistic regression analyses to control its effect.

**TABLE 3:** *F*-test for differences among regression slopes (*p*-value), Correlation coefficients (r), slopes (b), and *p*-values between Age and LZC variables by groups.

	Slope test	CN-no-DS		CN-DS		MCI-DS	
	*P*-value	r (b)	*P*-value	r (b)	*P*-value	r (b)	*P*-value
LF_LZC	0.033	0.638 (0.0007)	0.014	0.151 (0.0003)	0.608	−0.424 (−0.0012)	0.131
RF_LZC	0.025	0.667 (0.0008)	0.009	0.229 (0.0005)	0.431	−0.417 (−0.0010)	0.138
LP_LZC	0.003	0.536 (0.0014)	0.048	0.004 (<0.0001)	0.988	−0.626 (−0.0022)	0.017
RP_LZC	0.008	0.535 (0.0014)	0.049	0.087 (0.0002)	0.767	−0.566 (−0.0019)	0.035
LT_LZC	0.061	0.432 (0.0011)	0.123	0.036 (<0.0001)	0.903	−0.402 (−0.0017)	−0.424
RT_LZC	0.004	0.509 (0.0012)	0.063	0.047 (0.0001)	0.872	−0.424 (−0.0014)	0.131
LO_LZC	0.038	0.438 (0.0015)	0.117	0.204 (0.0008)	0.483	−0.494 (−0.0020)	0.072
RO_LZC	0.047	0.494 (0.0017)	0.072	0.227 (0.0010)	0.436	−0.434 (−0.0017)	0.121

CN, control; DS, Down syndrome; LF, left frontal; LO, left occipital; LP, left parietal; LT, left temporal; MCI, mild cognitive impairment; RF, right frontal; RO, right occipital; RP, right parietal; RT, right temporal; SD, standard deviation.

The analyses showed no relationship between sex and LZC values in any of the anatomical regions: (CN-no-DS group: *p* > 0.120 for all regions; CN-DS group: *p* > 0.578 for all regions; MCI-DS group: *p* > 0.519 for all regions).

### Logistic regression analyses

Two independent logistic regression models were estimated to discriminate between CN-DS and CN-no-DS groups, and between CN-DS and MCI-DS groups. In both models, the CN-DS group was defined as the reference category. As demographic analyses evidenced a group-dependent relationship between age and LZC values in certain regions and following Hosmer-Lemeshow’s suggestions (Hosmer et al., 2013) on “clinically relevant” variables, we considered age as a “fixed” variable to be included in all the multivariate models (see Table 4).

**TABLE 4:** *P*-values of the univariate regression models (LR for likelihood-ratio test).

	CN-DS vs. CN-no-DS	CN-DS vs. MCI-DS
	LR test *P*-value	LR test *P*-value
LF_LZC	0.068	0.010
RF_LZC	0.096	0.017
LP_LZC	0.009	0.148
RP_LZC	0.007	0.326
LT_LZC	0.139	0.108
RT_LZC	0.098	0.081
LO_LZC	0.272	0.835
RO_LZC	0.368	0.646

#### Control DS group vs. CN-no-DS

**FIGURE 3:**
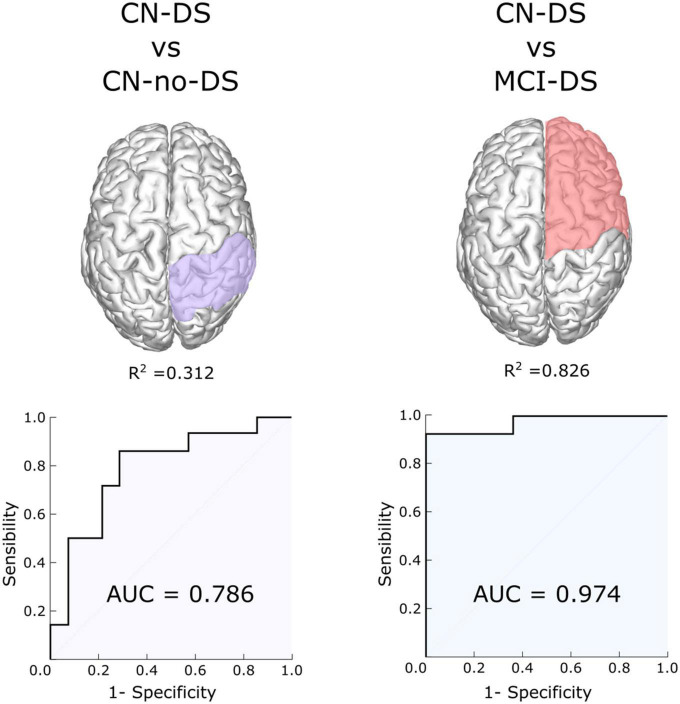
ROC curves of multivariate logistic regression models including AUC and Nagelkerke coefficient (*R*^2^). *R*^2^ represents that about 31% of the “variability” in the CN-DS vs. CN-no-DS comparison, and about 80% of the variability in the CN-DS vs. MCI-DS comparison is explained by the logistic models. AUC, Area Under the ROC Curve; CN, control; DS, Down syndrome; LZC, Lempel-Ziv complexity; MCI, mild cognitive impairment.

The final multivariate regression model, controlled for age, only contained the RP_LZC values (Likelihood ratio test = 7.309; *p* = 0.007). The Hosmer-Lemeshow goodness-of-fit statistic was 9.433 (*p* = 0.223) and the Nagelkerke-coefficient R^2^ was 0.312. The area under the ROC curve (AUC) was 0.786 (*p* = 0.010, 95% CI = [0.610; 0.962]). Both the sensitivity and specificity of the model were 71.4%, when a 0.50 cut-off point is adopted. The ROC curve is displayed in Figure 3.

#### Control DS group vs. MCI group

The final multivariate regression model, controlled for age, only contained the RF_LZC values (Likelihood ratio test = 5.248; *p* = 0.022). The Hosmer and Lemeshow goodness-of-fit statistic was 5.388 (*p* = 0.613), and the Nagelkerke-coefficient R^2^ was 0.826. The AUC was 0.974 (*p* < 0.001, 95% CI = [0.920; 1.000]). The sensitivity and specificity of the model were 92.9 and 85.7%, respectively, when a 0.50 cut-off point is adopted. The ROC curve is shown in Figure 3.

### Lempel-Ziv complexity and neuropsychological performance

Significant variables in the final logistic regression models were submitted to correlation analyses. Within the MCI-DS group, a significant positive correlation was found between RF_LZC values and *short-term verbal memory-learning_delayed*, *working memory* and s*hort-term verbal memory-learning*, while marginal correlations were observed with *praxis total* (Table 5). Similarly, RP_LZC exhibited a significant correlation with *short-term verbal memory-learning*_*delayed*; while *short-term verbal memory-learning* and *constructive praxis* showed slightly weaker but also significant correlations (Table 5). No significant correlations were found within the CN-DS group.

**TABLE 5:** Spearman correlation coefficients (r) and *p*-values between neuropsychological variables and LZC values within the MCI-DS group.

	RF_LZC		RP_LZC	
	r	*P*-value	r	*P*-value
Short-term verbal memory-learning_delayed (BT-ID)	0.757	0.003	0.779	0.002
Working memory (BT-ID)	0.588	0.039	0.437	NS
Short-term verbal memory-learning (BT-ID)	0.583	0.036	0.561	0.046
Constructive praxis (BT-ID)	0.131	NS	0.606	0.048
Praxis total (CAMCOG-DS)	0.532	0.061	0.440	NS

BT-ID, Barcelona Test-Intellectual Disability; CAMCOG-DS, Cambridge Cognitive Examination for older adults with Down’s Syndrome; DS, Down syndrome; RF, right frontal; LZC, Lempel-Ziv complexity; NS, not significant; RP, right parietal.

## Discussion

To the best of our knowledge, this is the first study using brain signal complexity to assist in the detection of MCI in DS. Overall, LZC mean values indicated that the MCI-DS group showed the lowest complexity scores, while the CN-DS group exhibited intermediate scores as the CN-no-DS group exhibited the highest values. However, results indicated that such a tendency had a regional specificity, as parietal scores differentiated CN-DS from CN-no-DS groups and frontal scores differentiated CN-DS from MCI-DS groups. The CN-no-DS group exhibited the expected pattern of significant increase of LZC as a function of age, while that pattern was “broken” in CN-DS and, especially, in MCI-DS. The combination of reduced LZC scores and a divergent trajectory of complexity evolution with age allowed the discrimination of MCI-DS patients with a high sensitivity and specificity.

The present results are not in disagreement with the fact that DS and AD share some basic pathophysiological signs with the AD-spectrum. Particularly, amyloid plaques and neurofibrillary tangles are present in almost all patients with DS by the age of 40 (Ballard et al., 2016), leading to a lifetime risk of developing dementia greater than 90% (Bittles et al., 2007; McCarron et al., 2017; Fortea et al., 2020). Regarding the incidence of MCI in DS, a very recent investigation (Oliver et al., 2022) reported that MCI was evident in approximately 20% of adults aged 40 and under, 40% aged 41–50, and 45% aged 51 and over. Considering the proved existence of AD neuropathology, most patients with DS and MCI aged above 40 might be labeled as “MCI due to AD” (Albert et al., 2011), and their associated neurophysiological features should resemble those observed in the general population. EEG studies using conventional analysis techniques indicated that DS patients with dementia show the typical features of AD, including increased power in delta and theta bands (Medaglini et al., 1997; Velikova et al., 2011; Salem et al., 2015). With respect to MCI, a recent MEG investigation (García-Alba et al., 2019) showed a widespread increase of theta activity in DS that was replaced by an augmented delta in the MCI stage. As these results mirrored the evidence observed in populations with typical development, we assumed that complexity changes in the MCI-DS group should follow the same logic.

Brain signal complexity in MCI cases without DS has been extensively investigated in EEG and MEG studies in recent years (Bruña et al., 2012; Maturana-Candelas et al., 2019; Shumbayawonda et al., 2020; Şeker et al., 2021; Ding et al., 2022). Most of these studies showed that patients with MCI had lower complexity than controls, but higher values than AD patients. Notably, none of these investigations explored the influence of age on complexity scores or its potential interaction with the neuropathological process. Age exerts a well-known influence on brain complexity (Anokhin et al., 1996; Meyer-Lindenberg, 1996; Yao et al., 2013), with a brisk increase from infancy to adolescence and a sustained linear increase at least until the sixth decade of life. This “normative” profile was complemented by Goldberger’s (Goldberger et al., 2002) approach to physiological complexity that assumes a breakdown with “aging and disease.” Fernández et al. (2012) demonstrated that the linear increase of complexity scores reaches a maximum by the age of 60, and after that, values slowly decrease until late senescence. Also, complexity tends to decrease in most of the investigated pathologies, but some examples exist of increased scores (for a review see Fernández et al., 2013a). This indicates that “disease” does not produce a mere reduction of the different markers of physiological complexity, but rather a disruption of its normal evolution with aging.

For instance, such disruption was demonstrated in attention deficit-hyperactivity disorder (Fernández et al., 2009), major depression (Méndez et al., 2012), and schizophrenia (Fernández et al., 2011a). When this approach was applied to patients with MCI (Fernández et al., 2010), complexity scores were not only lower as compared with controls, but also their evolution as a function of age failed to follow the expected trend. In addition, results evidenced the crucial role of reduced complexity values in parieto-occipital and frontal regions, accompanied by age effects to discriminate among groups. Current findings confirmed that the CN-DS, and especially the MCI-DS group, show abnormal patterns of complexity evolution with age, as well. According to the age-intervals of the sample, the scores of both groups should display a linear increase, but CN-DS cases exhibited an almost flat regression line, and MCI-DS patients displayed a clear negative association between age and complexity. Here it is important to note that the profile seen in MCI-DS patients is not identical to the observed in population with typical development. In Fernández et al. (2010), MCIs showed the “flat line” now detected in CN-DSs, while the negative correlation with age now detected in the MCI-DS group was seen in ADs. At that time results were justified by an abnormally accelerated aging process, a perspective that is controversial for AD in the general population but well-supported in DS. In fact, DS has been defined as a “progeroid” syndrome since patients with DS suffer from several age-associated disorders (particularly of the central nervous system) much earlier than euploid persons (Martin, 1978; Franceschi et al., 2019). This is a crucial aspect, as DS cases in our sample seemed to exhibit neurophysiological features that usually correspond to later stages of the neurodegenerative process. Such a perspective was also adopted by Babiloni et al. (2009) in a study performed in adolescents with DS. The spectral profile displayed by DS cases virtually paralleled the observed in early AD, although the sample included very young non-demented adults.

These facts might be partially explained by recent results reported by Nakamura et al. (2018). Most patients with DS aged above 40 show significant levels of amyloid deposition and this feature exerts a definite effect on the oscillatory activity of the brain. Nakamura et al. (2018) demonstrated that the typical profile of increased low-frequency activity in posterior regions, which was previously considered to be a signature of the AD-spectrum in EEG/MEG studies, was associated with general cognitive decline but is not specific because these changes could be observed in the absence of amyloid deposition. MCI individuals with confirmed amyloid positivity that progress to AD showed a pattern of increased delta activity in frontal regions. This key finding supports the classical notion of a posterior-to-anterior tendency of neurophysiological abnormalities observed with the progression of the disease (Jeong, 2004). Such posterior-to-anterior tendency also appears in our sample, as parietal sources discriminated CN-no-DS individuals from CN-DS cases, while frontal sources discriminated CN-DS from MCI-DS cases.

The next issue to be discussed is how these features are related to the complexity profiles detected in our DS patients, and to the abnormal evolution of the LZC values with age. A pattern of predominant delta activity, as it has been described in MCI-DS cases, reduces the frequency components of the brain signals, and therefore produces a significant decrease of complexity scores (Aboy et al., 2006). Of note, such increase of delta activity has been related to a cholinergic deficit. Classical studies revealed that AD patients showed a significant negative correlation between cholinergic activity in the CSF and delta power (Riekkinen et al., 1991), that can be experimentally reproduced by means of scopolamine infusions or lesion to the nucleus basalis of Meynert (Neufeld et al., 1994; Holschneider et al., 1997, 1999). This cholinergic deficit is also present in adults with DS, leading to the therapeutic use of cholinesterase inhibitors (Godridge et al., 1987; Sinai et al., 2017). An additional factor that has been associated with a reduction of brain signal complexity with aging and disease is the loss of connections (Sporns et al., 2000), especially of the excitatory type (Yao et al., 2013). AD was defined as a “disconnection syndrome,” and this characteristic is also present in DS. Recent investigations reported significant alterations in white matter integrity that were considered a core feature of the disease (Lee et al., 2016; Rosas et al., 2020). Fernández et al. (2011b) demonstrated that complexity and fractional anisotropy show a positive correlation, thus implying that the loss of white matter integrity due to a pathological process or to the effects of age is associated with a reduction of complexity values.

When analyzing the relationship of regional complexity values and neuropsychological variables, a significant association was observed in the MCI-DS group between RF_LZC, *short-term verbal memory-learning*, *verbal short-term memory-learning_delayed* and *working memory*; *total praxis* showed a marginal significance. RP_LZC values also showed a significant correlation with *short-term verbal memory-learning_delayed;* and a weaker but also significant correlation with *short-term verbal memory-learning and praxis constructive*. At the clinical level, executive dysfunction is characteristic of the prodromal stage of cognitive decline in DS (Rubenstein et al., 2020; Esteba-Castillo et al., 2022), which should be considered in the possible diagnosis of MCI in DS. In line with our results, previous neuroimaging and MCI-characterization research showed that the prefrontal lobe appears selectively affected in patients with DS before dementia (Pujol et al., 2018; García-Alba et al., 2019).

However, the correlations between memory-learning variables with frontal and parietal regions were less expected. In this regard, studies of recognition memory revealed that the parietal lobe plays a key function in memory processes (Berryhill and Olson, 2009; Gonzalez et al., 2015). More concretely, Krumm et al. (2017) found that the parietal lobe critically supports successful immediate and delayed target recognition memory, and that the ventral aspect of the parietal cortex and the medial temporal lobe may have complementary preferences for identifying targets and rejecting distractors, respectively, during recognition memory. In fact, a mnestic pattern in the MCI in DS is characteristic (Firth et al., 2018; García-Alba et al., 2019; Ramírez-Toraño et al., 2021; Esteba-Castillo et al., 2022). The third group of neuropsychological variables related to changes in complexity are praxis. The parietal lobes play a determining role in praxis (Kawai et al., 2013), providing the sensory maps that facilitate movement execution. More specifically, visuospatial functions have been associated with damage in the right temporo-parietal region and in the posterior occipital areas (Moore and Demeyere, 2022). In DS, the decline in visuospatial skills and motor coordination has been related in the years prior to clinical AD (Firth et al., 2018; Rubenstein et al., 2020).

It is worth noting several limitations of this study. First, the sample size of 42 individuals is relatively small. Also, age had a different distribution among groups, with the MCI-DS group showing an average older age when compared with the other two groups. Therefore, age effects were controlled in all the classification analyses. Additionally, due to the behavioral characteristics of the DS sample that prevented the evaluation with MRI, a T1-MRI template was used instead of the individual structural data, and this could diminish the spatial resolution in the source reconstruction process. Nevertheless, this procedure has been used successfully in previous works (García-Alba et al., 2019; Ramírez-Toraño et al., 2021). Despite these limitations, the present study still offers relevant information on the changes in brain signal complexity in adults with DS and their possible influence on cognitive performance. More importantly, results suggest that DS is a new example of a disease that breaks the normal evolution of complexity with age, and such basic feature might assist in the detection of MCI within this population.

## Data availability statement

The raw data supporting the conclusions of this article will be made available by the authors, without undue reservation.

## Ethics statement

The studies involving human participants were reviewed and approved by the Research Ethics Committee; Hospital Universitario de la Princesa, Madrid, Spain. The patients/participants provided their written informed consent to participate in this study.

## Author contributions

AF and FR-T contributed to writing, analyzing, and interpreting the data, and editing the manuscript. AF contributed to acquire the funding and supervised the study. RB, DA, and ES provided guidance regarding the complexity analysis, including scripts for the analysis, and helped to interpret the results. PZ performed the statistical analyses. SE-C and FMa surveyed the work and helped with the interpretation of the results. FMo was responsible for the recruiment of the sample and supervised the clinical diagnosis. JG-A and SE-C conceptualized the research, contributed to writing and interpretation, supervised the study, and acquired the funding. All authors contributed to the article and approved the submitted version.
